# Influence of the Results of Control of Intakes, Proteins and Anthropometry Nutritional Screening, Sarcopenia and Body Composition on the Clinical Evolution of Hospitalized Patients

**DOI:** 10.3390/nu16010014

**Published:** 2023-12-20

**Authors:** Elena Márquez Mesa, José Pablo Suárez Llanos, Patricia Mercedes Afonso Martín, Carla Brito Negrín, María García Ascanio, Samuel González González, Ignacio Llorente Gómez de Segura

**Affiliations:** 1 Endocrinology and Nutrition Department, Hospital Universitario Nuestra Señora de Candelaria, Ctra. Del Rosario 145, 38010 Santa Cruz de Tenerife, Spain; psuarezllanos@gmail.com (J.P.S.L.);; 2 Faculty of Health Sciences, University of La Laguna, Calle Padre Herrera, 38200 La Laguna, Spain

**Keywords:** disease-related malnutrition, CIPA, sarcopenia, EWGSOP2, hand grip strength, appendicular skeletal muscle index, body composition, phase angle, hospital stay, mortality

## Abstract

(1) Background: Hospital malnutrition and sarcopenia are common in inpatients and are associated with worse prognosis. Our objective is to determine the association of the positivity of CIPA (Control of Intakes, Proteins and Anthropometry) nutrition screening tool and sarcopenia and evaluate its prognostic implications (length of stay, readmissions and mortality) as well as different components of body composition. (2) Methodology: Cross-sectional single-center study and prospective six months follow-up for prognostic variables. On admission, CIPA and EWGSOP2 criteria were assessed. (3) Results: Four hundred inpatients, a median of 65.71 years old and 83.6% with high comorbidity, were evaluated. In total, 34.8% had positive CIPA and 19.3% sarcopenia. Positive CIPA and sarcopenia had worse results in body composition (fat mass (FM), fat-free mass (FFM) and appendicular skeletal muscle mass index (ASMI)) and dynamometry. Positive CIPA is significantly associated with worse prognosis (mortality (OR = 1.99), readmissions (OR = 1.86) and length of stay (B = 0.19)). Positive CIPA and sarcopenia combined are associated with a tendency to higher mortality (OR = 2.1, *p* = 0.088). Low hand grip strength (HGS) is significantly related to a higher length of stay (B = −0.12). (4) Conclusions: In hospitalized patients, malnutrition independently and combined with sarcopenia is associated with a worse prognosis but not body composition. Low HGS is related to a higher length of stay.

## 1. Introduction

Malnutrition is a poor prognostic factor for inpatients, but numerous research papers corroborate that nutritional intervention can improve the clinical evolution of hospitalized malnourished patients [1]. The development of the GLIM (Global Leadership Initiative on Malnutrition) criteria has made it possible to have a common strategy for nutritional evaluation. It is made up of two steps: first, a validated nutritional screening test is carried out, and then the nutritional evaluation itself, analyzing phenotypic and etiological criteria, including the evaluation of reduced muscle mass [2].

Hospital malnutrition is a frequent problem in patients admitted to a hospital. Prevalences ranging between 10% and 50% have been observed. In Spain, the multicenter PREDYCES study found that 23.7% of hospitalized patients were malnourished or at nutritional risk [3], while the seDREno study, using the GLIM malnutrition criteria, observed that 29.7% of hospitalized patients were malnourished [4].

A nutritional screening method called CIPA (Control of Intakes, Proteins and Anthropometry) was designed at Hospital Universitario Nuestra Señora de Candelaria (HUNSC) in Tenerife. In this tool, different items are evaluated: (a) decrease in intake < 50% in the first 72 h of admission; (b) plasma albumin < 3 g/dL; and (c) BMI < 18.5 kg/m^2^ or mid-upper arm circumference (MUAC) ≤ 22.5 cm (if the BMI cannot be determined). Positivity of at least one of these items translates into a positive CIPA nutritional screening and identifies the patient with malnutrition or at risk of suffering from it. Since 2015, it has been implemented in the HUNSC and has been evaluated by different validation, optimization and cost-effectiveness studies [5,6].

In addition, the importance of assessing body composition is being increasingly recognized. The European Working Group on Sarcopenia in Older People 2 (EWGSOP2) has established new criteria for the diagnosis of sarcopenia, evaluating muscle mass and muscle function [7]. These parameters can be measured in daily clinical practice by bioimpedance analysis (BIA) and hand grip strength (HGS), respectively. 

The BIA is the most widespread instrumental method in the study of body composition. It is a non-invasive, low-cost and easily accessible technique. The most frequently applied model to evaluate body composition is two-compartmental, dividing the body into fat mass (FM) and fat-free mass (FFM) that includes bone mineral content, extracellular water, intracellular water and visceral protein [8]. In the assessment of body composition, the BIA is based on the principle of the resistance that the body offers to an electric current, and the FFM can be estimated using predictive equations [9]. Different studies have shown that altered results of these items are associated with worse prognostic outcomes [10,11]. 

Dynamometry is a functional muscle strength assessment method that measures the isometric strength of the hand and forearm. It is a cheap and easy measurement to perform, so its implementation in clinical practice is simple. Furthermore, there are normality values with which to compare in numerous populations. Hand dynamometry tends to adequately reflect the body’s muscle strength and correlates well with the body lean mass determined by different techniques such as BIA, densitometry (DXA) and computed tomography (CT) and with analytical measures of inflammation such as the decrease in plasma albumin [12,13]. Likewise, HGS has clinical and prognostic value, being associated with greater morbidity and mortality, worse quality of life and functional limitations [14,15,16].

Loss of muscle mass and muscle function are common in inpatients, especially in older and malnourished ones, and have potentially serious adverse effects. Different studies have shown that the presence of sarcopenia was associated with a worse quality of life, higher readmission rate and mortality [17,18,19]. 

For this reason, it is important to detect malnourished patients early, or those at risk of malnutrition, as well as those with sarcopenia, in order to implement appropriate therapeutic measures to reduce the associated side effects and improve the prognosis. Therefore, we investigate whether the malnutrition or risk of malnutrition determined by the CIPA nutrition screening tool and/or the presence of sarcopenia determined by the EWGSOP2 criteria is associated with changes in body composition as well as worse prognostic evolution (death, length of stay and readmissions at six months). 

## 2. Materials and Methods

### 2.1. Type of Study and Ethical Aspects

Cross-sectional single-center study carried out in patients > 18 years old admitted in HUNSC evaluating the presence of malnutrition or risk of presenting it using de CIPA screening tool and sarcopenia determined by EWGSOP2 criteria and subsequent prospective follow-up of patients for up to six months. The ethics committee of HUNSC gave its approval to carry out this study on 17 December 2020 (project code CHUNSC_2020_105). The study was carried out in accordance with the requirements expressed in the Declaration of Helsinki [revision of Fortaleza (Brazil), October 2013] and the Laws and Regulations in force in Europe and Spain. The information sheet was delivered to the participating subjects. The investigator explained to the patient the objectives and procedures of the study and requested the signing of the informed consent form. Once the consent was signed, the researcher began the explorations and data collection necessary for the study. The investigator did not initiate any investigation corresponding to the study until the consent of the patient had been obtained.

### 2.2. Inclusion and Exclusion Criteria

The inclusion criteria included adult subjects of both sexes with a hospital stay of more than three days who were attached to one of the following departments: general surgery, internal medicine, vascular surgery, digestive system, hematology, nephrology, pneumology, oncology, neurology, traumatology or cardiology. The exclusion criteria included subjects not eligible for CIPA nutritional screening at the HUNSC with a prognosis of hospital stay of less than or equal to three days; admission to a department with a low incidence of malnutrition (ophthalmology, dermatology, obstetrics …); pediatric patient or critical care unit and palliative care; or patients already receiving artificial nutritional treatment. Patients with edemo-ascitic overload were also excluded. Written informed consent was requested from patients who met all the inclusion criteria and none of the exclusion criteria, and in the case of minors or disabled patients, that of their parents or legal guardians was collected.

### 2.3. Collected and Analyzed Data

The malnutrition screening that is usually used in the hospital (CIPA) was performed, to which the EWGSOP2 criteria were added. The evaluation of malnutrition and functionality was carried out after three days of hospital stay. The scores of both were recorded together with the data collection via the clinical history. For the CIPA test, BMI, albumin levels and percentage of decreased intake were recorded. Positivity of at least one of these items was considered a positive CIPA nutritional screening result: (a) decrease in intake < 50% in 72 h; (b) plasma albumin < 3 g/dL; and (c) BMI < 18.5 kg/m^2^, MUAC ≤ 22.5 cm (if the BMI could not be determined) [20].

For the EWGSOP2 criteria, muscle mass and function were determined by BIA and HGS, respectively, and for de the diagnosis of sarcopenia, it was necessary that both items were diminished. Body composition was estimated by electrical bioimpedance (BIA 101^®^ Akern Anniversary, Akern SRL, Pontassieve, Florence, Italy) using electrical values to determine appendicular skeletal muscle mass (ASM). Raw measurements produced by the device were used along with the Sergi equation for ASM estimation in elderly patients (>65 years) [21] and the Kyle equation in patients between 18 and 65 years [22]. ASM index (=ASM/height^2^) values below 7 kg/m^2^ in men and 5.5 kg/m^2^ in women were considered as low muscle mass [7]. HGS was measured using a validated dynamometer Jamar^®^ (JLW Instrumets, Chicago, IL, USA); the patient was seated with the arm adducted at the side, with the elbow flexed to 90° and the forearm in a mid-prone position. Hand grip duration had to be of at least 3 s with the dominant hand, and the maximum strength of three repeated grips was used as the test score. Values under 27 kg in men and 16 kg in women were considered abnormal [7].

Together with the usual work protocols and data depending on the pathology under treatment, the variables collected were age, sex, cause of admission, comorbidity (Charlson comorbidity index (CCI)) and functionality. Subsequently, the sample of patients with a positive CIPA result received therapeutic interventions according to the usual protocol [20]. The patients were followed up for the study of prognostic factors that also were recorded: length of stay, readmissions in the next 30 days and mortality in the following 6 months. Patients were included in the period between February 2021 and April 2023.

### 2.4. Statistical Analysis

Qualitative variables were summarized as frequency distribution, and normally distributed quantitative variables as mean ± standard deviation (SD). The continuous, non-normally distributed variables were summarized as median and interquartile range (IQR). To assess the skewness of quantitative variables, a graphical inspection of histograms and box plots, together with quantile-quantile normality plots, was performed. For the analysis, a new variable was generated based on the combination of the positive results of sarcopenia and/or malnutrition (normal, CIPA positive, Sarcopenia positive and CIPA+ Sarcopenia positive). Qualitative variables were compared with the Pearson chi-square test. The comparison of normally distributed quantitative variables between two groups was performed using the Student’s *t*-test or analysis of variance (ANOVA) for more than two groups. 

The relationship of outcome variables (6-month mortality and readmission for 30 days) with the diagnosis of malnutrition and/or sarcopenia and body composition variables was assessed using binary logistic regression. For the outcome variable length of stay, a linear regression model was fitted. As the length of stay was not normally distributed, this data was log-transformed. Each model was adjusted by age, sex, CCI and department of admission. Statistical significance was assumed as *p* < 0.05. All analyses were performed using SPSS 26.0 (IBM Corp., Armonk, NY, USA).

## 3. Results

### 3.1. Characteristics of the Sample

A total of 400 patients who met the inclusion criteria during the study period and agreed to participate were recruited for the study. The most frequent admission departments were Digestive (13.8%), Traumatology (13.5%), Internal Medicine (12%), Pneumology (11.5%) and Neurology (10.3%). A percentage of 72.5% of the admissions were in a medical service and 27.5% in a surgical one. In total, 51.5% of the patients were male, the mean age was 65.71 ± 14.69 years and 83.6% had a CCI > 3, which is considered indicative of high comorbidity. Table 1 shows the baseline clinical characteristics and body composition.

### 3.2. Malnutrition and Sarcopenia Screening and Diagnosis

In total, 34.8% presented a positive CIPA, determining malnutrition or risk of suffering from it. A percentage of 20.5% presented plasma albumin < 3 g/dL, 15.8% decrease in oral intake < 50% and 5.8% BMI < 18.5 kg/m^2^. The CIPA was positive for presenting one altered item in 28.5% of the patients, two in 5.3% and three items in 1%. The parameters that were the most frequent cause of the CIPA positive result were plasma albumin < 3 g/dL (14.8%) and a decrease in oral intake < 50% in the first 72 h of admission (10%), both without alteration of the other items. 

Probable sarcopenia was observed in 62.5% of the patients with low HGS. Of the patients, 24.8% had low muscle mass by ASMI. Finally, sarcopenia was confirmed in 19.3% of the patients according to the EWGSOP2 criteria.

The combination of positive CIPA and sarcopenia occurred in 11% of the patients. Table 2 shows the characteristics of the patients based on the diagnosis of malnutrition or risk of malnutrition, and sarcopenia. Patients with sarcopenia and positive CIPA were older, had worse results in body composition (low BMI, HGS, muscle mass, FFM and muscle function) and had higher comorbidity.

### 3.3. Association between Prognostic Clinical Outcomes and CIPA Results, Sarcopenia and Body Composition

A mortality of 17.3% of the total sample was observed at 6 months, 7.5% of early readmission and a median stay of 14 (8–24) days. 

**Figure 1:**
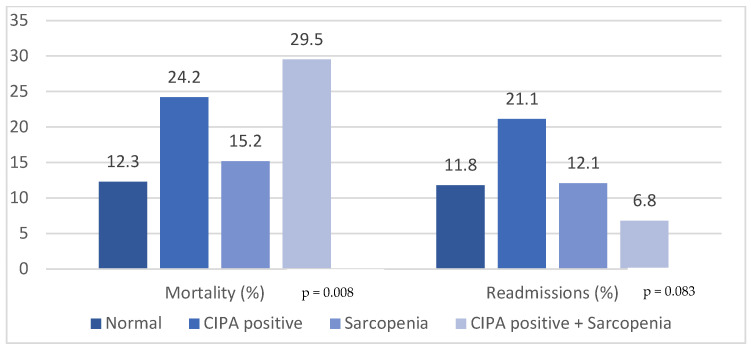
Percentage of mortality and readmissions by groups.

Positive CIPA alone and also a positive CIPA with sarcopenia were associated with higher mortality (24.4% and 29.5%, respectively) than normal patients (12.3%); *p* = 0.008. However, patients with a diagnosis of sarcopenia alone did not present higher mortality than patients without it and negative CIPA. Regarding early readmission rate (<30 days), a trend toward significance was observed with a higher readmission rate in the CIPA positive group vs. the negative group (21.1% vs. 11.8%; *p* = 0.083) (Figure 1).

An analysis of the relationship between other body composition variables and worse prognosis was performed, but no significant differences were observed regarding mortality or readmissions (Table 3).

Table 4 shows the results of the multivariate analysis of the relationship between the body composition variables and the diagnosis of malnutrition and/or sarcopenia with the outcome variables. These results were adjusted for age, sex, CCI and admission service (medical/surgical).

The CIPA-positive group had a higher mortality risk (OR = 1.99; *p* = 0.043). This was also observed in the CIPA positive and sarcopenia group, with close to statistical significance (OR = 2.1; *p* = 0.088). An increase in early readmissions rate was observed in the CIPA group, also close to statistical significance (OR = 1.8; *p* = 0.073), with no differences observed in the rest of the variables. A longer length of stay was observed in the CIPA-positive group (B = 0.19; *p* = 0.04). Also, a significant decrease in length of stay was observed as HGS increased (B = −0.012; *p* = 0.015) (Table 4).

## 4. Discussion

Our study evaluated the clinical prognostic value of malnutrition (or risk of presenting it via the CIPA nutrition screening tool), the presence of sarcopenia, and different body composition components. 

A prevalence of malnutrition or risk of it of 34.8% was detected, similar to that described in previous studies with this nutritional screening tool, 35.8% in no surgical patients [23] and 35.4% in surgical patients [5]. This prevalence is slightly higher than described in the PREDYCES study, 23.7% with Nutritional Risk Screening (NRS-2002) [3] and more similar to the 29.7% described in the seDREno study with the GLIM criteria [4]. However, we must take into account that in the PREDYCES study, the prevalence of malnutrition in the group of patients over 70 years of age increased to 37%. This could be related to the average age of our sample, close to 70 years, as well as the inclusion of other markers of malnutrition, such as albumin. 

The clinical evolution of patients detected as malnourished or at risk of malnutrition was worse than in patients with negative nutritional screening, presenting higher mortality and average length of stay and a trend toward a higher rate of early readmissions. This data is consistent with the previous results obtained in other studies in which CIPA has been used as the nutritional screening tool. CIPA detected that surgical patients had a greater risk of mortality during hospitalization (5% vs. 0%, *p* = 0.006), higher median length of stay (21 days [IQR 14–34 days] vs. 14.5 days [IQR 9–27 days], *p* = 0.002) and rate of early readmissions (25.3% vs. 8.2%, *p* < 0.001) [5]. In other studies, such as PREDyCES, it was also observed that malnutrition increased hospital stay (11.5 ± 7.5 versus 8.5 ± 5.8 d; *p* < 0.001) as well as costs [3]. More recently, the EFFORT Trial has shown that intensive nutritional treatment during hospitalization allows a 21% reduction in serious adverse effects that include mortality, admissions to the intensive care unit, readmissions after 30 days, major complications, functional impairment and mortality (OR = 0.65 (0.47–0.91); *p* = 0.011) [24]. These data reveal the importance of detecting malnutrition and its early management.

The sample analyzed had a high rate of comorbidities, being representative of the population of developed countries with a high rate of polymorbidity that is associated with a higher rate of complications, making an early evaluation of malnutrition and sarcopenia important [25].

The prevalence of sarcopenia was 19.3%, similar to that described in previous studies. Ballesteros et al. [18] evaluated the presence of sarcopenia in 200 hospitalized patients, presenting 33% of them with probable sarcopenia and 22.5% confirmed sarcopenia on admission, increasing to 53.3% at discharge. Cerri et al. [19] described the presence of sarcopenia in 21.4% of hospitalized patients with malnutrition or risk of malnutrition. The GLISTEN (Gruppo Lavoro Italiano Sarcopenia—Trattamento e Nutrizione) determined that 34.7% of 600 hospitalized elderly people presented sarcopenia at admission. This higher prevalence could be related to an older sample of patients (mean age 81.0 ± 6.8 years) [26].

Sarcopenia itself has been shown to be a negative prognostic factor in multiple pathologies. It increases the risk of falls and fractures, impairs the ability to perform activities of daily living, is associated with cardiac disease, respiratory disease and cognitive impairment, leads to mobility disorders and contributes to lowered quality of life, loss of independence or need for long term care placement and death [7]. Ballesteros et al. [18] found that patients with sarcopenia had a worse prognosis with a worse quality of life, higher readmission rate (OR = 2.25) and mortality (OR = 8.16). They independently analyzed the prognostic implications of HGS and muscle mass, finding that patients with higher HGS had a higher quality of life, fewer readmissions and less mortality adjusted for age, sex and comorbidities but not with low muscle mass alone. Also, the GLISTEN group [17] reported that patients with dynapenia had a longer hospital stay. These results are consistent with ours, in which we have observed that patients with altered HGS have a longer average stay and a trend toward higher mortality, but we have not observed an association of confirmed sarcopenia with worse prognostic evolution. This could be related to the difficulty in determining muscle mass since the pathologies themselves, as well as the treatments used in hospitalized patients (fluid therapy, hydroelectrolyte replacement and depletive therapies), can alter the results obtained via BIA. Other more accurate methods could be used, such as DXA, CT or magnetic resonance imaging (MRI), but their limited availability and the emission of ionizing radiation limit their use in clinical practice. The standardization and use of muscle ultrasound in the evaluation of sarcopenia could be of interest and is currently being developed in different populations [26]. On the other hand, dynamometry can be implemented easily and at a low cost, presenting a good correlation with body muscle strength. Numerous studies have described its association with higher mortality and complication rates in different pathologies, reinforcing its role as a prognostic marker [27], being recommended in the latest expert consensus on morphofunctional assessment of malnutrition related to the disease [28]. However, it must be evaluated whether the established cut-off points are the most appropriate. Some studies, such as that of Westbury et al., use more lax cut-off points that allow the identification of a greater prevalence of sarcopenia while maintaining a strong association with mortality [29].

Furthermore, in recent years, interest has grown in the study of different components of body composition, such as FM and FFM, as well as ASMI, being these two last parameters of phenotypic criteria of malnutrition in the GLIM malnutrition criteria [2]. Body composition has been studied in many pathologies, but not so much in heterogeneous hospitalized patients of different ages. In the study by Ji et al. [11], they found that reduced muscle mass determined by ASMI in cancer patients was associated with worse survival. Cereda et al. [10] analyzed the FFM index (FFMI) in a cohort of cancer patients, observing that patients with a decreased FFMI had higher mortality and lower quality of life. In our study, an association of worse results in the body composition values of the different compartments with a higher prevalence of malnutrition and sarcopenia was evident. However, it was not observed that patients with altered body composition data had a worse prognostic outcome. 

The results obtained in our study, showing a worse clinical evolution in patients with decreased muscle function (determined by HGS) but not in patients with low muscle mass, could be related to the fact that the decrease in muscle strength can appear even before changes in the measurements of muscle mass are observed. Furthermore, this alteration in functionality could be more related to the alteration of muscle quality than to the quantity. Roberti et al. [30] found that the amount of intermuscular fat deposits induces alterations of muscle quality without alterations of muscle quantity influencing the patient prognosis. Pereira et al. [31] did not identify a correlation between sarcopenia and the rate of adverse surgical outcomes in patients with early-stage breast cancer. Also, we must take into consideration that the use of predictive equations is necessary to estimate the different body compartments. Normally, these equations have been developed in healthy populations, but the age of the patients, the different pathologies, as well as the ethnic origin, can affect these estimates. This is why the evaluation of raw physical parameters is increasingly used in clinical practice, and its inclusion in the evaluation criteria for sarcopenia and malnutrition has been suggested [32].

As a limitation of this study, it should be noted that it is a single-center study with a limited number of patients, so the data must be extrapolated with caution to the general population. No functional tests were performed that would allow for the grading of the severity of sarcopenia. It was not recorded which patients received nutritional or rehabilitation therapy, so it was not possible to evaluate whether those who were treated had a better prognostic outcome.

## 5. Conclusions

In summary, we found that patients with malnutrition or at risk of suffering from it, as well as those who associate sarcopenia with malnutrition, have worse clinical outcomes. These groups of patients also present worse results in FM, FFM and ASMI. Special attention should be paid to muscle functionality, as, like in other works, low HGS appears to be a marker of a worse clinical prognosis. This is an interesting issue on the one hand because this evaluation is easy to perform, and on the other because muscle functionality impairment appears before the muscle mass is affected, so it can be an early marker.

Therefore, we consider early detection of malnutrition and sarcopenia (and especially muscle function) to be of great importance in order to early predict patients with worse clinical evolution.

## Figures and Tables

**Table 1:** Baseline clinical characteristics and body composition data of the included patients.

	n = 400 Mean (SD)
Age (years)	65.71 (27.23)
Sex (% men)	51.5
CCI	7.63 (5.33)
BMI (kg/m^2^)	27.23 (6.39)
HGS (kg)	19.17 (10.64)
ASMI (kg/m^2^)	7.36 (1.68)
FFM (kg)	52.22 (11.58)
FM (kg)	21.98 (13.45)
Albumin (g/dL)	3.54 (0.62)

SD: standard deviation. CCI = Charlson comorbidity index. BMI = body mass index. HGS = hand grip strength. ASMI = appendicular skeletal muscle mass index. FFM = fat-free mass. FM = fat mass.

**Table 2:** Characteristics of the patients depending on the diagnosis of malnutrition or risk of malnutrition by CIPA screening and/or diagnosis of sarcopenia by EWGSOP2 criteria.

	Normal	Positive CIPA	Sarcopenia	Positive CIPA + Sarcopenia	*p*
n (%)	228 (57)	139 (34.8)	77 (19.3)	44 (11)	-
Age (years) *	63.45 (14.51)	65.77 (15.51)	74.3 (10.85)	70.84 (12.89)	<0.01
Sex (% men)	50.99	45.3	57.9	63.6	0.204
BMI (kg/m^2^) *	29.12 (6.03)	27.41 (6.06)	23.06 (3.03)	20.12 (4.09)	<0.01
Admission service (% surgical)	29.4	27.4	27.3	18.2	0.508
CCI *	6.93 (5.07)	8.42 (5.66)	8.28 (5.04)	9.02 (5.68)	0.023
Albumin < 3 g/dL (%)	3.81 (0.45)	3.03 (0.63)	3.57 (0.44)	3.19 (0.58)	<0.01
Low HGS (%)	48.2	65.2	100	100	<0.01
Low muscle mass (%)	6.6	7.4	100	100	<0.01
FFM (kg) *	54.7 (11.04)	53.66 (12.03)	43.97 (7.46)	42.04 (6.81)	<0.01
FM (kg) *	24.93 (14.3)	20.67 (12.03)	17.68 (8.6)	12.79 (8.75)	<0.01

* data expressed as mean and standard deviation. CCI = Charlson comorbidity index. BMI = body mass index. HGS = hand grip strength. FFM = fat-free mass. FM = fat mass.

**Table 3:** Association of body composition variables and prognostic evolution (readmissions and mortality).

	No Readmissions	Readmissions	*p*	No Mortality	Mortality	*p*
BMI (kg/m^2^)	27.27 (6.58)	26.98 (5.03)	0.755	27.2 (6.43)	26.33 (6.18)	0.202
HGS (kg)	19.23 (10.94)	18.74 (8.47)	0.757	19.54 (10.71)	17.37 (10.19)	0.132
ASMI (kg/m^2^)	7.32 (1.7)	7.67 (1.5)	0.150	7.38 (1.69)	7.28 (1.63)	0.654
FFM (kg)	51.97 (11.75)	53.84 (10.47)	0.270	52.34 (11.57)	51.67 (11.75)	0.663
FM (kg)	22.38 (13.76)	19.49 (11.02)	0.143	22.38 (13.77)	20.09 (11.67)	0.197

Data expressed as mean and standard deviation (SD). BMI = body mass index. HGS = hand grip strength. ASMI = appendicular skeletal muscle mass index. FFM = fat-free mass. FM = fat mass.

**Table 4:** Risk of worse prognostic evolution (mortality, readmissions, length of stay) with respect to diagnostic groups (malnutrition and/or sarcopenia) and body composition variables.

	Mortality (<6 Months)		Readmissions (<30 Days)		Length of Stay (Log-Transformed)	
	OR_a_ (IC 95%)	*p*	OR_a_ (IC 95%)	*p*	B_a_ (IC 95%)	*p*
Normal	Ref		Ref		Ref	
Positive CIPA	1.99 (1.02–3.91)	0.043	1.86 (0.94–3.65)	0.073	0.19 (0.01;0.38)	0.040
Sarcopenia	1.01 (0.33–3.08)	0.9	1.16 (0.35–3.79)	0.805	0.21 (−0.08;0.49)	0.159
Positive CIPA + Sarcopenia	2.10 (0.90–4.92)	0.088	0.43 (0.12–1.58)	0.205	0.19 (−0.05;0.45)	0.126
BMI	0.98 (0.94–1.03)	0.466	0.99 (0.95–1.04)	0.901	−0.01 (−0.02;0.003)	0.130
HGS	0.97 (0.93–1.01)	0.097	0.98 (0.94–1.02)	0.372	−0.012 (−0.02;−0.002)	0.015
ASMI	0.94 (0.78–1.13)	0.510	1.10 (0.92–1.31)	0.295	0.009 (−0.04;−0.057)	0.727
FFM	0.97 (0.95–1.01)	0.115	1.00 (0.97–1.04)	0.798	−0.005 (−0.01;0.003)	0.208
FM	0.99 (0.97–1.01)	0.483	0.98 (0.96–1.01)	0.141	−0.004 (−0.01;0.01)	0.126

OR_a_ (IC); B_a_ (IC). BMI = body mass index. HGS = hand grip strength. ASMI = appendicular skeletal muscle mass index. FFM = fat-free mass. FM = fat mass.

## Data Availability

Data supporting the reported results are available upon request.
